# Primary pleural squamous cell carcinoma misdiagnosed as localized mesothelioma: a case report and review of the literature

**DOI:** 10.1186/1749-8090-8-50

**Published:** 2013-03-17

**Authors:** Xiao-Ming Lin, Chuang Chi, Jun Chen, Yu Liu, Peng Li, Yi Yang

**Affiliations:** 1 Department of Cardiac and Thoracic Surgery, The First Affiliated Hospital of Wenzhou Medical College, 2#, Fuxue Lane, Wenzhou, 325000, China; 2 Department of Clinical Skills Experiments Center, Wenzhou Medical College, Wenzhou, China; 3 Department of Intensive Care, The First Affiliated Hospital of Wenzhou Medical College, Wenzhou, China; 4 Department of Pathology, The First Affiliated Hospital of Wenzhou Medical College, Wenzhou, China

**Keywords:** Primary pleural squamous cell carcinoma, Pleural mesothelioma, Solitary fibrous tumor, Surgical resection

## Abstract

Primary pleural squamous cell carcinoma is very rare, and there is a lack of experience in the diagnosis and treatment of this condition. An asymptomatic 75-year-old man was referred to us after a right pleural nodule was found on computed tomography during a routine health examination. He underwent surgery for his pleural tumor twice over the following 2 years. Histopathological examination eventually led to a diagnosis of primary pleural squamous cell carcinoma.

## Background

Primary pleural squamous cell carcinoma (SCC) is very rare [1]. A review of the literature did not find any recently reported cases. As patients are generally asymptomatic in the early stage and computed tomography (CT) shows local pleural thickening or small nodules, primary pleural SCC is easily misdiagnosed as localized mesothelioma. The histopathological features of these two tumors are also similar.

Unfortunately, pleural SCC and localized mesothelioma have different oncological characteristics. Localized mesothelioma usually has a good prognosis [2], but pleural SCC is characterized by malignant tumor growth with invasion of the surrounding tissues and organs, and metastasis. Delays in correct diagnosis and appropriate treatment therefore have serious clinical consequences and result in poor prognosis.

## Case presentation

**Figure 1:**
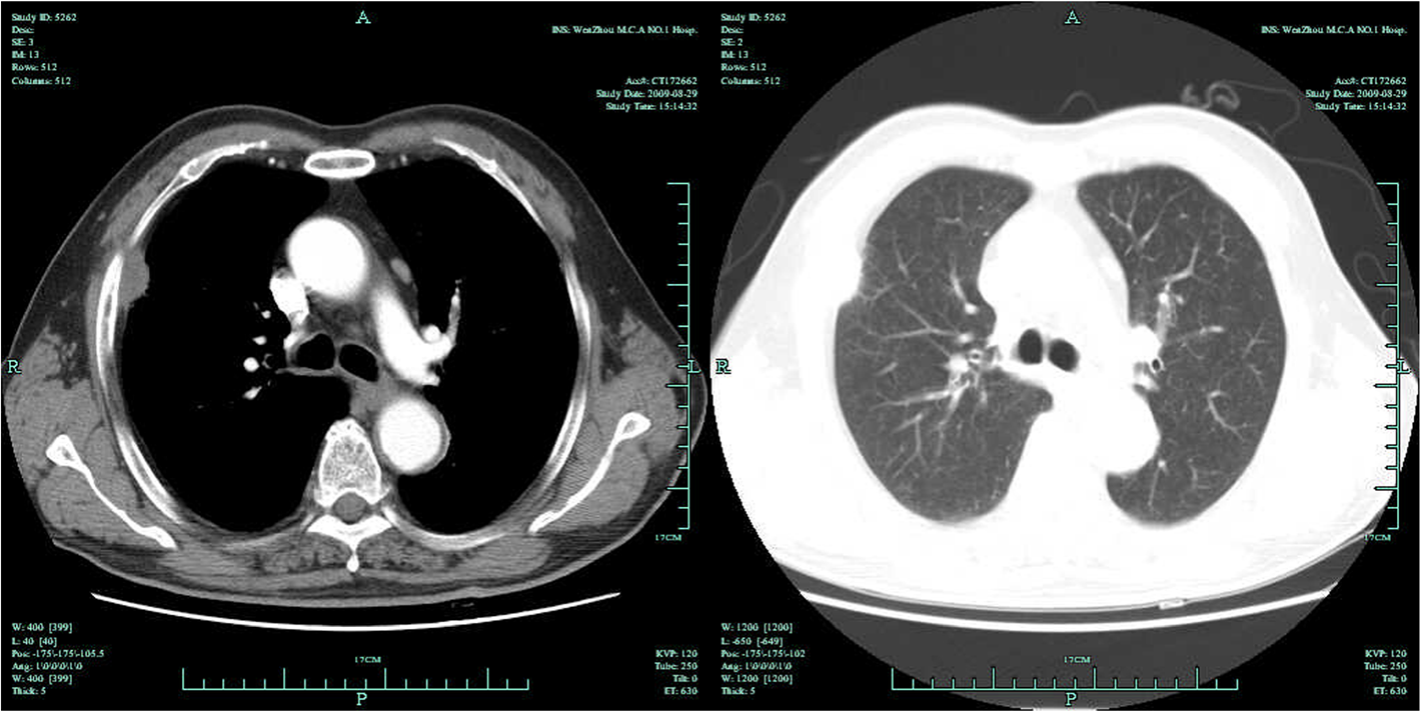
First chest CT scan on 29 August, 2009.

**Figure 2:**
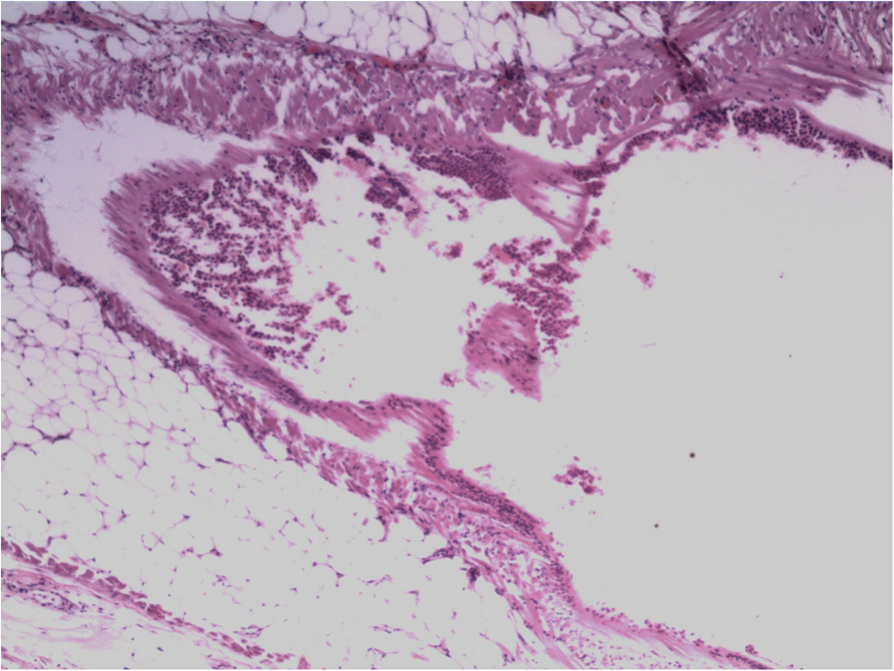
Pathological specimen from the first operation (hematoxylin and eosin staining, × 40).

In August 2009, a 75-year-old man (Chinese, ethnic Han) was referred to our clinic after a right pleural nodule was found on chest CT during a routine health examination. He was asymptomatic, and physical examination was unremarkable. Chest CT showed a soft tissue nodule with homogeneous enhancement arising from the right pleura, measuring 31 × 15 mm. The nodule had clearly demarcated margins and there was no evidence of invasion into the adjacent ribs. There was no enlargement of mediastinal lymph nodes (Figure 1). Video-assisted thoracic surgery (VATS) revealed a smooth nodule on the parietal pleura at the fourth intercostal space, measuring 35 × 20 mm and not adherent to the adjacent lung. The nodule was completely resected. Postoperative histopathological examination revealed fibrous tissue hyperplasia with inflammatory cell infiltration and mesothelial cell proliferation, and a diagnosis of pleural mesothelioma was made (Figure 2). The patient recovered quickly and was discharged from hospital on the seventh day after surgery.

**Figure 3:**
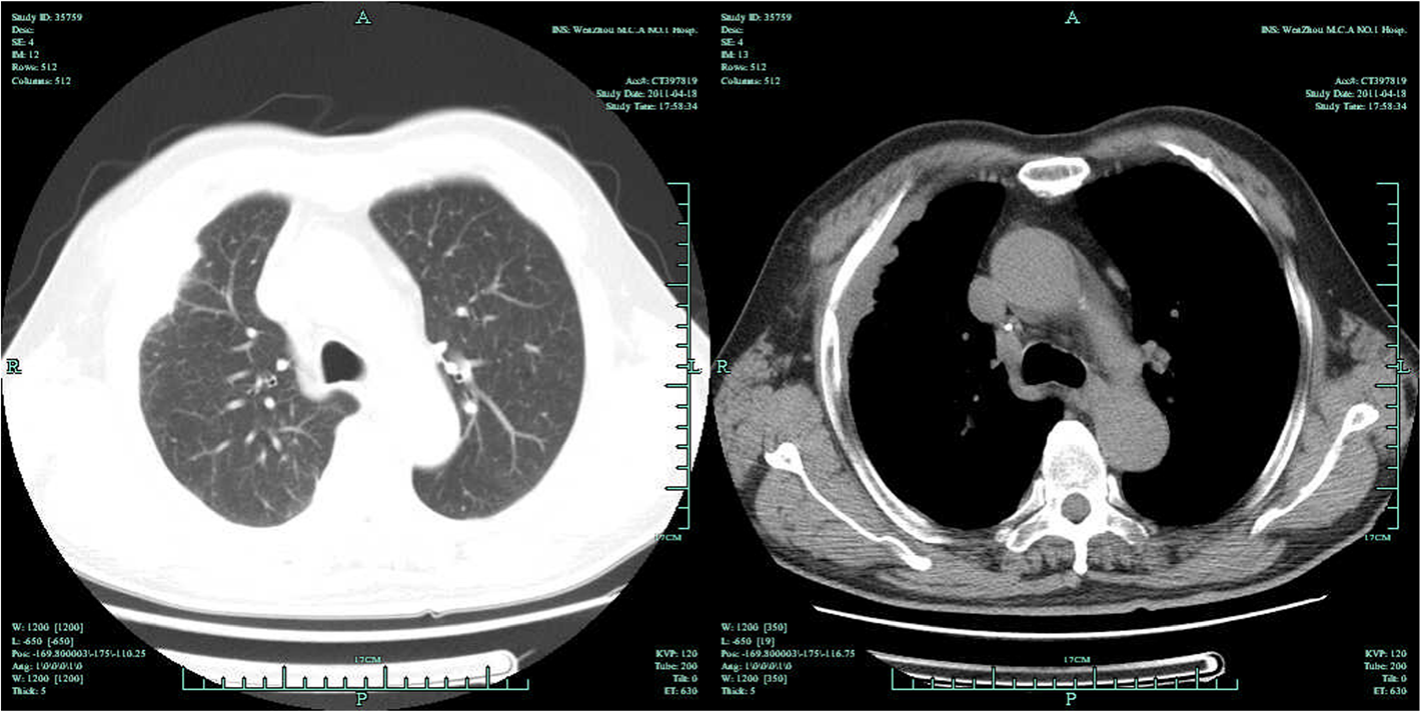
Second chest CT scan on 18 April, 2011.

In April 2011, he presented again with a 2-month history of right-sided chest pain, cough, sputum production, fatigue and weight loss. Physical examination revealed right chest wall tenderness and a hard, poorly delineated mass at the fourth intercostal space. Chest CT revealed right anterolateral pleural thickening, a soft tissue shadow in the adjacent chest wall, and clear lung fields with no enlargement of the mediastinal lymph nodes (Figure 3). Based on imaging findings, he was thought to have recurrence of pleural mesothelioma. Preoperative tumor antigen testing detected an elevated squamous cell carcinoma antigen (SCCA) level of 5.7 μg/l (normal value < 1.5 μg/m1).

**Figure 4:**
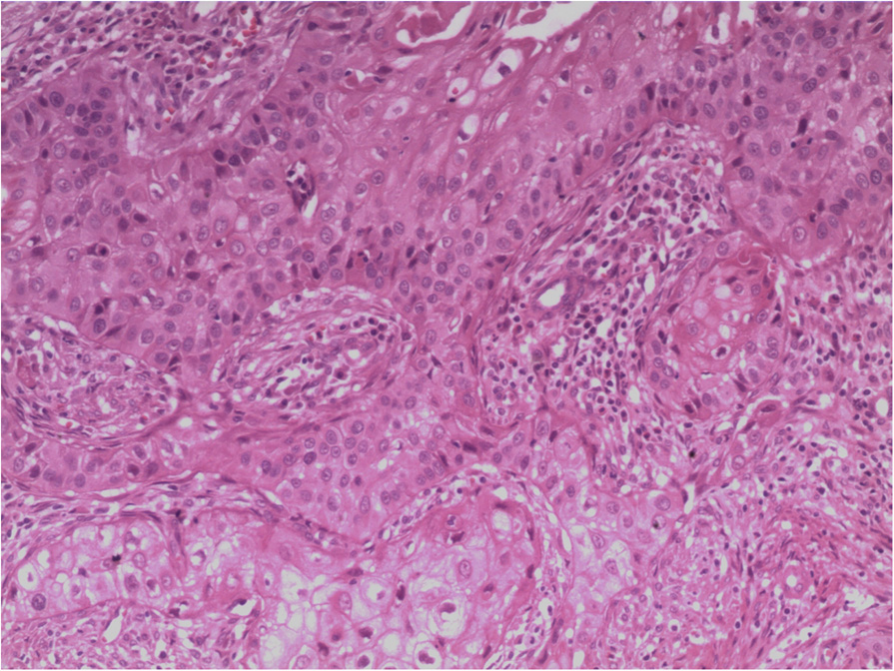
Pathological specimen from the second operation (hematoxylin and eosin staining, × 100).

**Figure 5:**
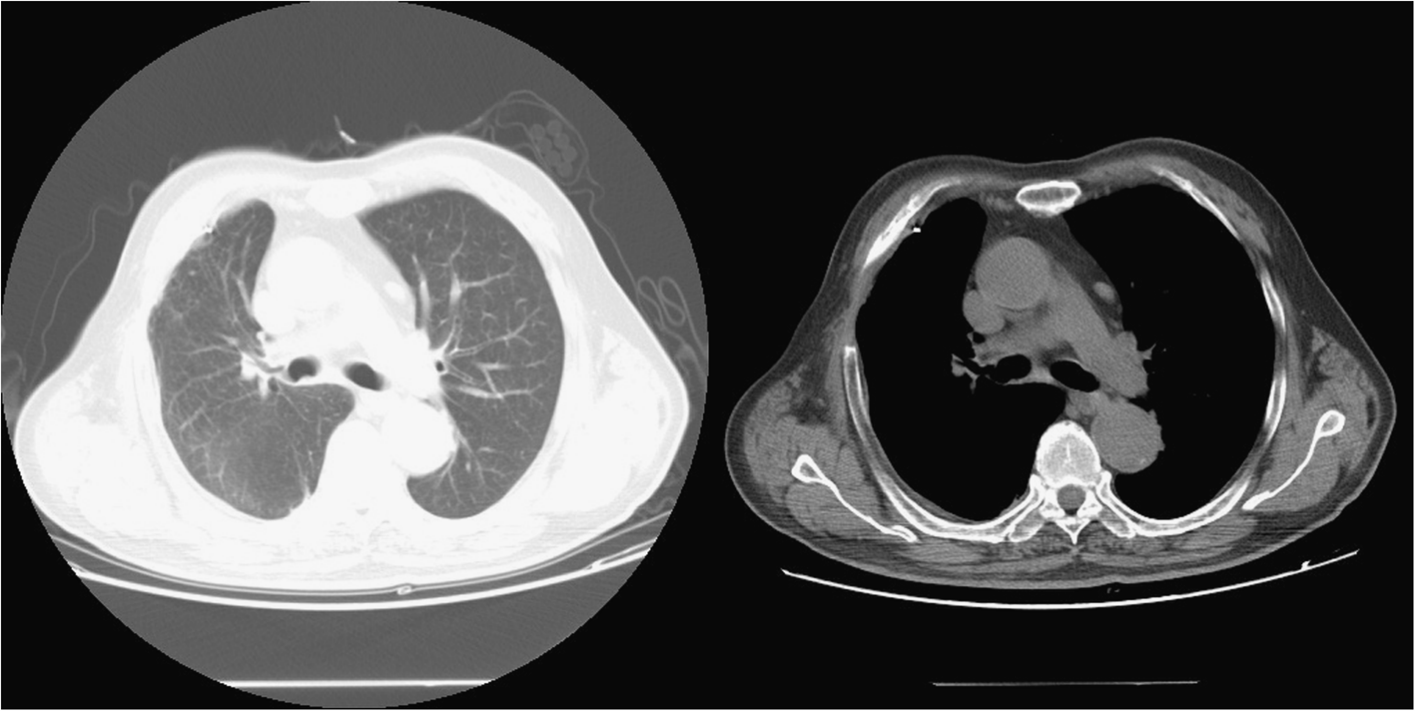
Third chest CT scan on 18 October, 2012.

We performed right thoracotomy through the fourth intercostal space. The tumor in the subcutaneous tissues measured 5 × 4 × 4 cm, and was continuous with a pleural lesion measuring 7 × 8 × 8 cm, which had poorly demarcated margins and had invaded the upper lobe of the right lung. Intraoperative biopsy of the pleural tumor revealed primary pleural SCC. En bloc resection of the tumor was performed, including the chest wall, pleura, part of the right upper lobe, part of the fourth and fifth ribs, and tissues of the intercostal space. Postoperative histopathological examination revealed areas of pleomorphic tumor cells with large nuclei and reduced cytoplasm. These features were consistent with stage I SCC (Figure 4). The patient recovered well. One month after operation, he returned to the hospital and received a 50Gy local radiation therapy. In October, 2012, his chest CT showed that the operation area was in good condition (Figure 5).

### Discussion

The majority of pleural tumors are metastases from primary tumors in organs such as the lung or breast [3]. Primary pleural tumors are rare, and are usually diffuse or localized mesotheliomas [4]. Primary pleural SCC is a particularly rare pleural malignancy which has seldom been reported.

Because of similar morphology, early primary pleural SCC may be misdiagnosed as localized mesothelioma, especially solitary fibrous tumor (SFT) of the pleura. However, these two conditions have different progression and prognosis. In order to avoid misdiagnosis and inappropriate treatment, it is necessary to distinguish between the two carefully.

SFT of the pleura is also uncommon, accounting for 4% of all pleural neoplasms [5]. SFT is not associated with asbestos exposure and usually has a good prognosis [2]. According to reviews by Briselli et al. [6] and England et al. [7], 12% to 33% of SFTs of the pleura were considered to be malignant according to their pathological characteristics. Due to the extreme rarity of primary pleural SCC, its incidence is not described in the literature, and the cause of this disease is unclear. We performed a literature search and found that SCC arising from the pleura had been reported in a few patients with chronic postpneumonectomy bronchopleural fistulas [1]. Chronic pleural inflammation may therefore be one of the causes of primary pleural SCC.

Early pleural SCC that has not invaded the surrounding structures is usually asymptomatic. As the disease progresses, some atypical symptoms may appear, such as chest pain, cough, sputum production, weakness and weight loss. Early pleural SFT is also asymptomatic [8,9], but disease development may result in some typical manifestations. Hypertrophic osteoarthropathy is observed in about 20% of cases, which may result from production of hyaluronic acid or hepatocyte growth factor by the tumor [10-12]. Hypoglycemia occurs in 2% to 4% of cases and is thought to be due to production of insulin-like growth factor II (IGF-II), which lowers the blood glucose level and impairs the growth hormone counter-regulatory response to hypoglycemia [12]. Hemorrhagic pleural effusions may occur in advanced pleural SCC, and serous pleural effusions may occur in about 10% of cases of pleural SFT [9]. These different symptoms of the two diseases may help to differentiate them.

The lack of specific features makes it difficult to differentiate between early primary pleural SCC and early pleural SFT on preoperative imaging examinations such as chest radiography and computed tomography [8,10]. These imaging modalities can show the locations of the lesions but cannot distinguish between benign and malignant lesions. Whole-body 18 F-deoxyglucose (FDG)-positron emission tomography (PET) has become a popular imaging modality in recent years, and can accurately differentiate benign from malignant tumors, as FDG uptake is very high in malignant tumors [13,14].

Preoperative biopsy is important for the diagnosis of pleural tumors. The reported rate of accurate diagnosis on CT-guided fine needle aspiration is about 45% in some series [10,11]. However, transthoracic Tru-Cut needle biopsy can obtain more tissue for histological and immunohistochemical analysis, and may be a better choice [5]. When the nature of pleural nodules is difficult to determine, such invasive examinations may be useful.

Several tumor markers may also be helpful for determining the type of pleural tumor. P63 is always negative in cases of mesothelioma and almost always positive in cases of SCC [15]. Calretinin is considered to be one of the best markers for differentiating between mesothelioma and other thoracic neoplasms, as it is strongly and diffusely positive in all types of mesothelioma, and generally negative or only focally positive in other types of neoplasm [16]. A number of studies have reported that cytokeratin 19 fragment (CYFRA 21–1) is the most sensitive biomarker for SCC, and another biomarker of the cytokeratin family had also demonstrated good diagnostic ability [17]. Although SCC antigen (SCCA) has a lower sensitivity than CYFRA 21–1, it has higher specificity for SCC [17]. Our patient tested positive for SCCA.

Surgery is the preferred method of treatment for pleural nodules. Cardillo et al. [10] reported that 87% of SFTs originated in the visceral pleura and only 13% in the parietal pleura. Most SFTs have well-demarcated margins and do not invade the surrounding structures, and complete surgical resection is not difficult. Early primary pleural SCC is also treated by surgical excision. It is unknown how often SCC originates in the parietal pleura, because of the small number of reported cases.

VATS is the preferred procedure for excision of pleural nodules because it is minimally invasive. The surgical treatment of choice is local excision with intraoperative assessment of the surgical margins [10]. If tumor-free surgical margins cannot be obtained using VATS, the procedure should be converted to open thoracotomy [9-11]. For some parietal pleural nodules with unclear edges which are suspicious for malignancy, intraoperative frozen section examination should be performed to help decide whether to perform extended resection to reduce the risk of recurrence and improve prognosis [9-11]. Postoperative adjuvant therapy for pleural malignancy has seldom been reported in the literature.

Some reports have suggested that CT should be performed every 6 months for the first 2 years after excision of pleural tumors to monitor for recurrence. Most recurrences of malignant pleural tumors occur within 24 months of the initial resection. Recurrence rates after complete resection were reported to be 8% in patients with benign pleural SFT and 63% in patients with malignant pleural SFT [18]. Further observation is required to determine whether patients with primary pleural SCC require the same follow-up after resection as those with pleural SFT.

For recurrent pleural SFT, repeat surgical resection may be the first choice of treatment, and is associated with good cure rates [19]. If recurrent pleural tumors are inoperable, then local radiotherapy and systemic chemotherapy should be given, although the optimal regimens have not been established [20]. Recent reports indicate that treatment with ifosfamide and doxorubicin may be effective. Observation of further cases is needed to determine whether the radiotherapy and chemotherapy regimens used for recurrent lung SCC are also effective in cases of primary pleural SCC.

## Conclusion

Primary pleural SCC is very rare. Because of the lack of clinical experience, early primary pleural SCC is easily misdiagnosed as localized mesothelioma, leading to delayed or inappropriate treatment. However, SCC can be diagnosed by preoperative investigations such as FDG-PET, measurement of tumor markers, and transthoracic Tru-Cut needle biopsy. The prognosis of this disease is not clear at present.

## Consent

Written informed consent was obtained from the patient for publication of this Case report and the accompanying image. A copy of the written consent is available for review by the Editor-in-Chief of this journal.

## Abbreviations

SCC: Squamous cell carcinoma; CT: Computed tomography; VATS: Video-assisted thoracic surgery; SCCA: Squamous cell carcinoma antigen; SFT: Solitary fibrous tumor.

## Competing interests

The authors declare that they have no competing interests.

## Authors’ contributions

YY wrote the manuscript. CC, YL and XML performed the surgery. PL carried out the pathological examinations. XML and JC were involved in the final editing. All authors read and approved the final manuscript.
